# Unmanned Aerial Vehicles for High-Throughput Phenotyping and Agronomic Research

**DOI:** 10.1371/journal.pone.0159781

**Published:** 2016-07-29

**Authors:** Yeyin Shi, J. Alex Thomasson, Seth C. Murray, N. Ace Pugh, William L. Rooney, Sanaz Shafian, Nithya Rajan, Gregory Rouze, Cristine L. S. Morgan, Haly L. Neely, Aman Rana, Muthu V. Bagavathiannan, James Henrickson, Ezekiel Bowden, John Valasek, Jeff Olsenholler, Michael P. Bishop, Ryan Sheridan, Eric B. Putman, Sorin Popescu, Travis Burks, Dale Cope, Amir Ibrahim, Billy F. McCutchen, David D. Baltensperger, Robert V. Avant, Misty Vidrine, Chenghai Yang

**Affiliations:** 1 Department of Biological and Agricultural Engineering, Texas A&M University, College Station, Texas, 77843, United States of America; 2 Department of Soil and Crop Sciences, Texas A&M University, College Station, Texas, 77843, United States of America; 3 Department of Aerospace Engineering, Texas A&M University, College Station, Texas, 77843, United States of America; 4 Department of Geography, Texas A&M University, College Station, Texas, 77843, United States of America; 5 LASERS Laboratory, Department of Ecosystem Science and Management, Texas A&M University, College Station, Texas, 77843, United States of America; 6 Department of Mechanical Engineering, Texas A&M University, College Station, Texas, 77843, United States of America; 7 Texas A&M AgriLife Research, Texas A&M University, College Station, Texas, 77843, United States of America; 8 USDA-Agricultural Research Service, Aerial Application Technology Research Unit, 3103 F&B Road, College Station, Texas, 77845, United States of America; New Mexico State University, UNITED STATES

## Abstract

Advances in automation and data science have led agriculturists to seek real-time, high-quality, high-volume crop data to accelerate crop improvement through breeding and to optimize agronomic practices. Breeders have recently gained massive data-collection capability in genome sequencing of plants. Faster phenotypic trait data collection and analysis relative to genetic data leads to faster and better selections in crop improvement. Furthermore, faster and higher-resolution crop data collection leads to greater capability for scientists and growers to improve precision-agriculture practices on increasingly larger farms; e.g., site-specific application of water and nutrients. Unmanned aerial vehicles (UAVs) have recently gained traction as agricultural data collection systems. Using UAVs for agricultural remote sensing is an innovative technology that differs from traditional remote sensing in more ways than strictly higher-resolution images; it provides many new and unique possibilities, as well as new and unique challenges. Herein we report on processes and lessons learned from year 1—the summer 2015 and winter 2016 growing seasons–of a large multidisciplinary project evaluating UAV images across a range of breeding and agronomic research trials on a large research farm. Included are team and project planning, UAV and sensor selection and integration, and data collection and analysis workflow. The study involved many crops and both breeding plots and agronomic fields. The project’s goal was to develop methods for UAVs to collect high-quality, high-volume crop data with fast turnaround time to field scientists. The project included five teams: Administration, Flight Operations, Sensors, Data Management, and Field Research. Four case studies involving multiple crops in breeding and agronomic applications add practical descriptive detail. Lessons learned include critical information on sensors, air vehicles, and configuration parameters for both. As the first and most comprehensive project of its kind to date, these lessons are particularly salient to researchers embarking on agricultural research with UAVs.

## Introduction

### Demand for Increased Agricultural Productivity

To address the food and fiber needs of a world population increasing from 7.1 billion to a predicted 9.6 billion by 2050 [1], crop production per unit area must be increased dramatically while soil and other natural resources are conserved to maintain our ecosystem [2, 3]. Agricultural research has greatly improved crop yields over the last century; e.g., U.S. corn (*Zea mays* L.) yield increased from 1.76 Mg ha^-1^ in 1900 to 10.6 Mg ha^-1^ in 2015 [4, 5] through conventional plant breeding and changes in agronomic practices [6], but gains in many crops have slowed [7, 8]. Restoring yield advances to necessary levels while protecting our natural resources is achievable, but new methods must be brought to bear. Two emerging technological fields provide promise: high-throughput phenotyping and precision agriculture.

The concept of field-based high-throughput phenotyping involves using autonomous vehicles with numerous sensors to automatically collect large amounts of data on plant variability across genetic lines. Advances in DNA sequencing have greatly improved genotyping efficiency, so the lack of efficient phenotyping ability has become a bottleneck to dissecting the genetics of quantitative plant traits [9]. Breeding programs spend tremendous effort manually collecting routine phenotypic data on segregating populations such as plant height, plant population, flowering time and yield [10]. Automated data collection systems can more quickly collect large volumes of crop phenotypic data at the plant or row scale. Data collection speed is critical to surveying large numbers of subplots at a singular growth stage. High-throughput phenotyping thus places challenges on spatial and temporal sensing resolution, speed, flexibility and cost of autonomous sensing systems.

The concept of precision agriculture involves using data on the spatial variability of soil and crop characteristics to optimize the amount and timing of field applications of inputs like seed, fertilizer, and irrigation. Required data can be collected by proximal sensors on field equipment or by remote sensing from airborne vehicles or satellites. This practice has been developing for over 20 years, but recent improvements in positioning technologies and sensors have provided the means for highly precise optimization.

Satellite and aerial remote sensing offer advantages for data collection in agricultural fields and have been used to monitor soil properties [11], weed infestation [12], crop abiotic stress [13], biotic stress [14–16], yield and biomass levels [17, 18]. Numerous studies have demonstrated that remote sensing can be useful, but most have been “one-off” projects to demonstrate the technology and stopped short of developing routine methods such that the technology becomes useful and common across disciplines. Furthermore, high-resolution data are essential to both high-throughput phenotyping and full utilization of precision agriculture. Only recently have autonomous aerial systems become available that can provide centimeter-level resolution.

### Development and Use of Unmanned Aerial Systems

Growing civilian application of unmanned aerial systems (UAS) offers opportunities to meet the needs of today’s agricultural production and research. UAS consist of an unmanned aerial vehicle (UAV), its payload, and a ground control station (GCS) for mission planning and flight control. In the last ten years, the cost and technological barriers for UAV flights have been lowered, and users now include university researchers, crop consultants and even a few farmers. Compared with other remote sensing platforms, UAS allow lower flight altitude, lower operational cost and complexity, less dependence on weather conditions, higher spatial resolution, and shorter revisit time [19, 20]. UAS also complement ground-based sensing platforms for increasing throughput and frequency of data collection efforts while obviating the requirement for good soil conditions in order to enter farm fields.

Fixed-wing and rotary-wing are the main two types of UAVs used, although there are others (e.g., blimps) (Table 1). Both have distinct advantages and disadvantages for field-based agricultural applications. Rotary-wing UAVs can take off and land vertically, eliminating the need for runways. The autopilots for rotary-wing vehicles routinely have automatic takeoff and recovery capability as well as a “return home” capability that ensures the vehicle reliably returns to the launch point. Their ability to hover close to crops to obtain imagery or collect data makes them attractive for highly detailed plant measurement. But because the electric motor must directly generate all of the required lift to keep the vehicle in the air, they tend to have small payload (sensor) capacity because most of the capacity is taken up by batteries. They also fly at low speeds and have a low endurance (flight duration between battery charges). Because of all these factors they have a more limited coverage area. Lift on fixed-wing UAVs is generated by the wings during forward flight so the motor and therefore the batteries are not directly responsible for producing lift. Thus much longer flight endurance and flight speeds are possible compared to rotary-wing UAVs, and the payload capacity is typically much larger. However, fixed-wing UAVs must always fly at airspeeds above their stall speed, which can sometimes result in demanding requirements on the sensor configurations to generate desired data. Finally, while only some fixed-wing UAVs have an automatic recovery capability at the present time, most if not all that are suitable for field-based agricultural applications will have this capability in the near future.

**Table 1:** Comparison of different types of UAV platforms.

Configuration	Takeoff/Landing	Hover	Payload (kg)	Endurance (minutes)	Autonomy	Airspeed (m s^-1^)
Fixed-wing	conventional	no	up to 14	up to 60+	partial/full	up to 22+
Multirotor	vertical	yes	up to 2.5	up to 15	full	up to 11
Electric helicopter	vertical	yes	up to 5	up to 15	partial/full	up to 12
Gas helicopter	vertical	yes	up to 20	up to 15	partial/full	up to 20
Blimp	vertical	yes	up to 4.5	up to 60+	Partial/full	up to 2.5

### Sensing Technology

UAVs are commonly outfitted with customizable sensor payloads for agricultural data collection. Miniaturized, light-weighted airborne sensors have been developed to meet the limited payload capability of small UAV platforms. Multiple types of cameras and other sensors are now available, and they are beginning to be used in measuring not only spectral but also morphological information, including plant height estimation and canopy surface profiling. Two approaches are currently used to extract morphological information. One is LiDAR (*Light Detection and Ranging*) [21], which uses pulsed light from a laser to measure distance from the sensor to ground objects so their positions can be mapped. LiDAR beams can also penetrate through crop canopy openings and return information about internal canopy structure, density, and the ground surface. The other approach is Structure-from-Motion (SfM) photogrammetry based on images taken from multiple perspectives [22] as a UAS flies over a field. Consumer-level digital cameras provide low-cost high-resolution images that can be used for SfM and measurement of some plant phenotypes including population, height, lodging, flowering time and yield [23–26].

Aside from morphology, spectral reflectance or radiance can be good indicators of plant vigor, abiotic and biotic stresses, biomass, yield potential and/or soil properties. Multispectral (commonly 3 to 6 spectral bands from 0.4 to 1.0 μm) and thermal cameras (commonly in the 7 to 14 μm range) have been adapted for small UAS applications: monitoring crop vigor and coverage [27, 28], detecting weeds in crop fields [29, 30], estimating yield and biomass [31–33], and detecting crop water stress [27, 31, 34]. Digital color cameras can be adapted to detect one or a few broad near-infrared (NIR) bands [35]. For continuous narrow-band spectral measurement over a wide spectral range, hyperspectral cameras (tens to hundreds of spectral bands) have been miniaturized for UAS but still require extra space and payload capacity.

### Necessity of Interdisciplinary Collaboration

UAV-based remote sensing in agriculture has recently become an active research specialization with demonstrated success in a few targeted projects, but an operational framework for routine collection of this kind of data in close partnership with field researchers has not been extensively investigated. Moreover, few studies have integrated widely diverse expertise involving agronomic researchers and breeders, air vehicle and sensor experts, remote sensing and data analysis experts, as well as end users to ensure that actionable data can be collected in a consistent and routine way.

**Fig 1:**
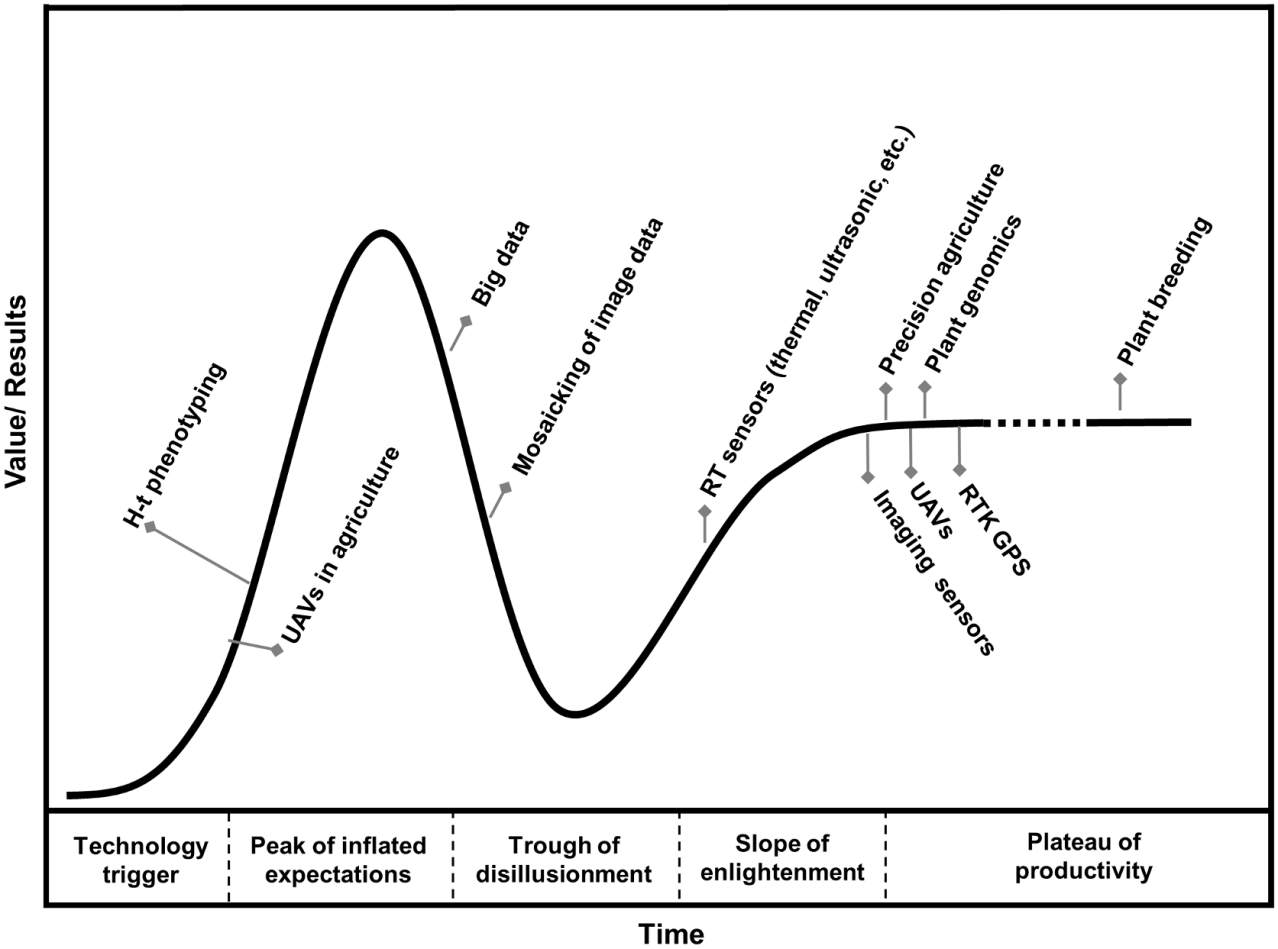
Gartner hype cycle cartoon of the subjective value and development stage of various technologies discussed here. Most of the base technologies are mature and productive, but the integration of all of these technologies is new and likely over-hyped.

Combining experts from across such a variety of disciplines can result in new and better solutions, but it also has the potential for miscommunication and unrealistic expectations. A Gartner hype cycle cartoon (Fig 1) demonstrates the development stages of various technologies used in this research field. UAS applications for crop phenotyping and agronomic research are expected to be a growing area of interest that builds on individual technologies (UAVs, sensors, analytical and data management methods), each with their own knowledge basis. Furthermore, expensive and specialized equipment is needed that would make comprehensive research in this field impractical for any one researcher or research group. The practical aspects of such a large interdisciplinary project constitute a challenging undertaking that is yet to be discussed in the literature.

In December of 2014, a group of Texas A&M University researchers across several disciplines and expertise areas converged to determine how the latest UAS technology could be used to improve agricultural research and production. This article reports on the details of our interdisciplinary workflow, from flight operations and sensing to data processing and analysis protocols, as well as case studies with a large number of lessons learned in the process of developing a comprehensive UAS remote-sensing effort of this sort.

### Objectives

The goals of this project were 1) to provide quick-turnaround, high-resolution, high-quality image data to a diverse group of agricultural researchers across numerous fields and plots on a large research farm; 2) to establish the workflow of data collection and processing as well as communication and coordination between experts that are required for such an endeavor; and 3) to develop methods for plant breeders and agronomists to incorporate UAS collected remote sensing data to improve their results and decision making ability.

## Materials and Methods

### Interdisciplinary Teams

**Fig 2:**
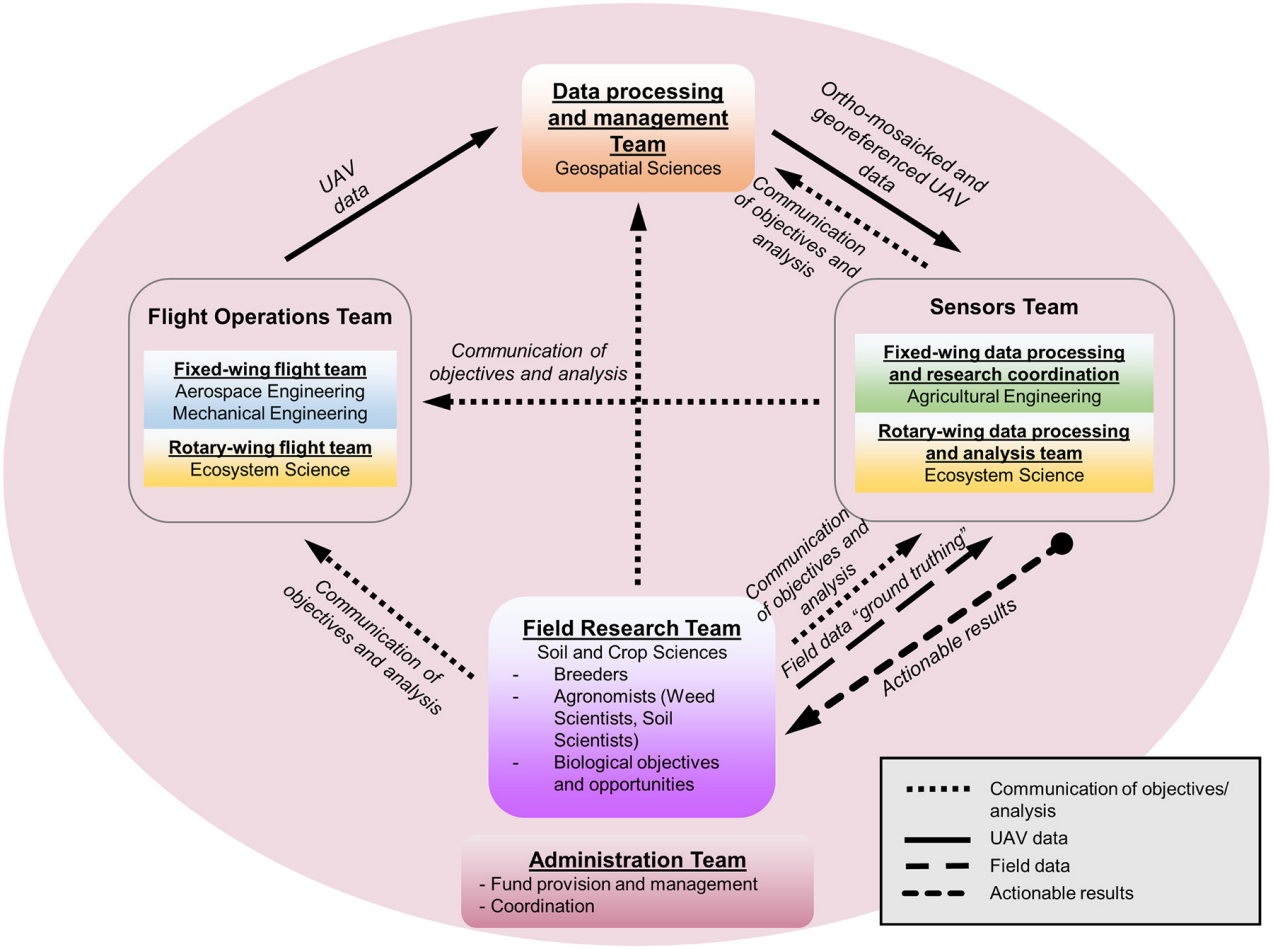
Generalization of the integration of teams and responsibilities in the TAMU-UAS project. Field researchers are end users and primarily involved in experimental design and ground-truthing data. Aerospace and mechanical engineers, and ecosystem scientists are primarily involved in raw UAS data collection. Geospatial scientists serve as a clearinghouse for the UAS data and also perform mosaicking—the stitching together of many small images to build one ortho-rectified and radiometrically seamless large image. Agricultural engineers are the nexus of the project, turning the UAS data into actionable results for the end users. The administration team provides and manages funds, facilitates meetings, and coordinates communications and initiatives.

The overall research group consisted of five teams (Fig 2) and roughly 40 scientists and engineers. The Administration team provided and managed funds, coordinated meetings and initiatives, and assisted and encouraged faculty members in garnering external funding. The Flight Operations team conducted UAV flights to provide remote-sensing data to the breeding and agronomic researchers. The Sensors team managed the sensors used onboard the UAVs, ensuring that the imagery received was of high quality, and worked with the breeding and agronomic researchers to develop analytic techniques. The Data Management team stored and managed the data and conducted pre-processing to geographically correct images and construct image mosaics. The Field Research team evaluated the data with respect to the ground-truth data they collected, and developed and used analytical tools in order to facilitate its breeding and agronomic research. The teams consisted of multiple investigators, some with very different objectives—most notably the Field Research team.

**Fig 3:**
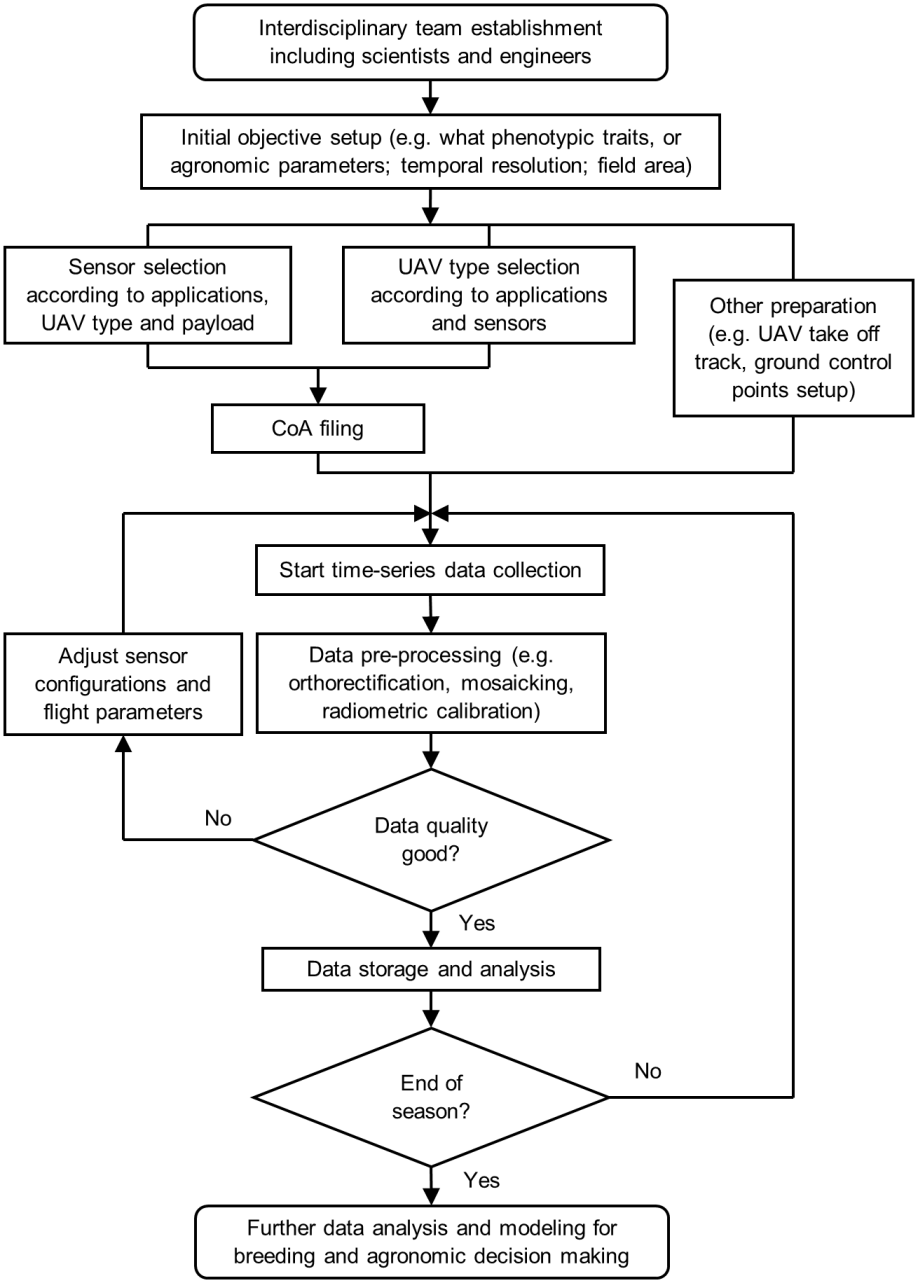
Workflow to start a UAV project for phenotyping and agronomic research, from interdisciplinary team establishment to decision making.

A certificate of authorization (COA) for this project was granted by the FAA to Texas A&M AgriLife Research on May 21, 2015, covering the agency’s entire Brazos Bottom research farm. Flights and experiments commenced June 6, 2015 and were carried out up to five times per week over selected field sites. Since each flight has a fairly large fixed cost, but a small marginal cost to add more acreage, we sought to collect as much data and involve as many field projects as possible during the first season. The system integration and configuration were improved over the course of the season to maximize data quality for data analysis and decision making (Fig 3).

### Experimental Site, Crops and Flight Route Packs

**Fig 4:**
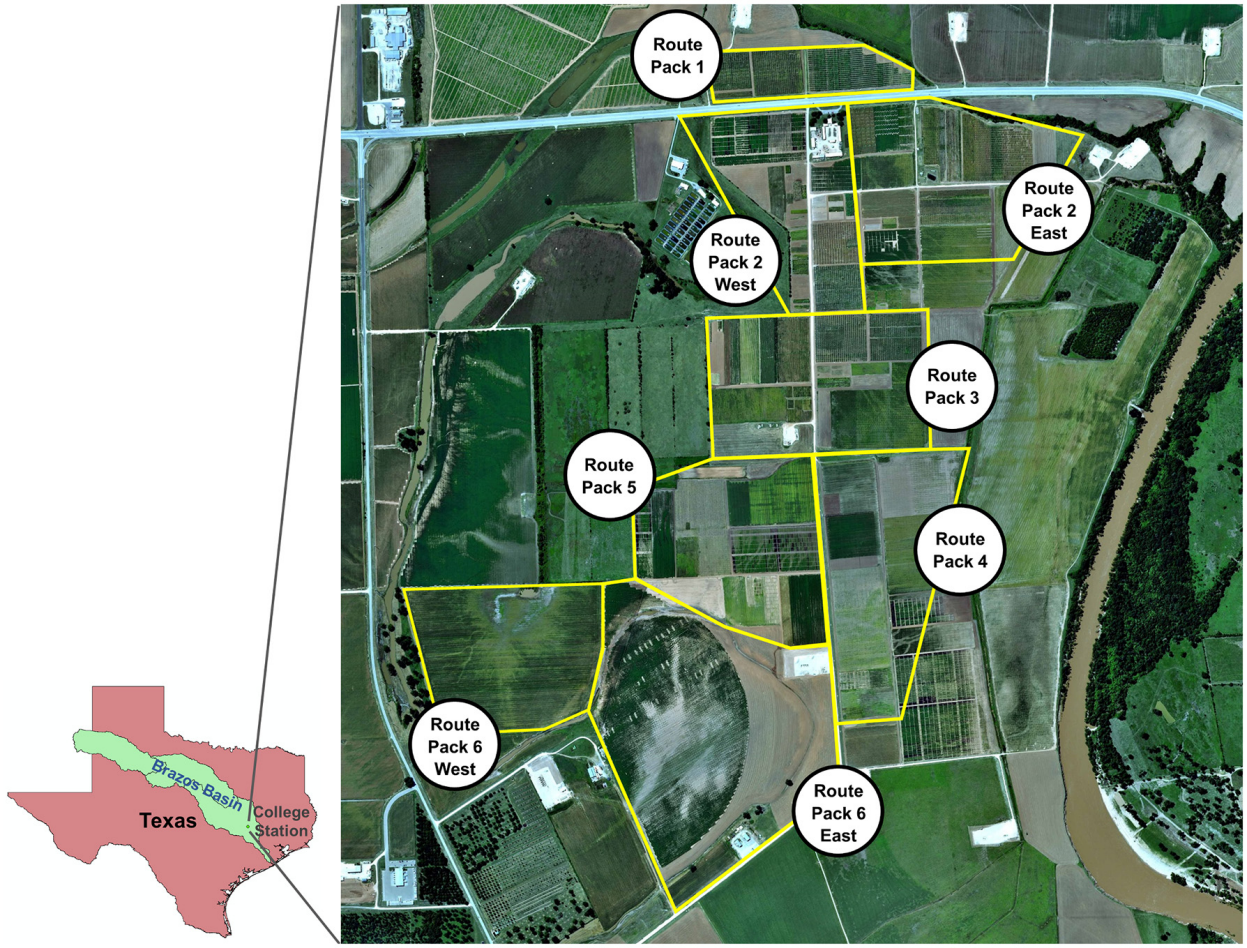
Experiment site—Brazos Bottom research farm. It was divided into route-packs (eight larger polygons in yellow) to be covered efficiently across different PI’s fields during an individual flight by fixed-wing UAVs. An individual flight of the multi-rotor UAV was based on a single field from a single researcher marked by smaller black polygons in the figure.

The experimental site was Texas A&M AgriLife Research’s Brazos Bottom Farm in Burleson County, Texas (headquarters at 30.549635N, 96.436821W). All research including weed control treatments was conducted under the authority of Texas A&M AgriLife Research. The farm consists of over 1300 ha, of which 568 are the crop fields and plots flown in this study. Major field crops including corn, cotton, sorghum and wheat are grown for breeding and agronomic research (Table 2). For fixed-wing UAS flights, which involve longer flight times, the farm was divided into contiguous groups of fields and plots called “route packs” that could be covered efficiently during an individual flight (Fig 4).

**Table 2:** Scale of the field breeding and agronomic research programs at the Texas A&M AgriLife Research’s Brazos Bottom research farm in College Station, summer 2015.

**Breeding Program**		
*Crop*	*Nursery (cases and evaluations)*	*Yield trials (measure yield)*
Corn	2.83 ha	10.1 ha
Cotton	8.09 ha	12.1 ha
Perennial grasses	1.21 ha	2.02 ha
Sorghum	4.05 ha	8.09 ha
Wheat	2.83 ha	4.05 ha
**Agronomic Programs**		
Soil and canopy properties	30 ha	
Weed management	10 ha	
Water use efficiency on cotton and corn	10 ha	

### UAV Platforms

**Fig 5:**
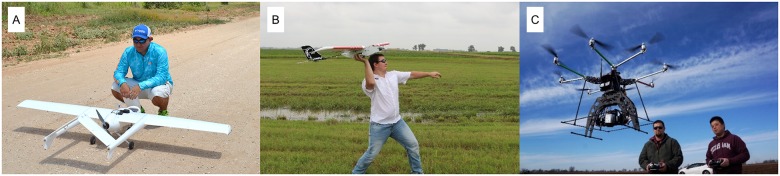
External physical characteristics of UAVs used in this study. (A) RMRC Anaconda fixed-wing vehicle. (B) PrecisionHawk Lancaster fixed-wing vehicle. (C) TurboAce X88 octocopter with autopilot computer interface (left), multiple battery options (middle), and navigation and gimbal transmitters (right). The individuals appearing in these figures gave written informed consent (as outlined in PLOS consent form) to publish these pictures.

Two fixed-wing UAVs and one rotary-wing UAV were operated by three separate flight teams (Table 3). The Anaconda (ReadyMadeRC, Lewis Center, Ohio, USA) (Fig 5a) is an electric-powered fixed-wing air vehicle designed with the conventional twin-tailboom pusher-propeller configuration. This air vehicle was the principal air vehicle for conducting research to develop protocols and test sensors. A 9-channel JR/Spektrum 2.4GHz direct-sequence spread spectrum radio was used for manual flight control during takeoff and landing, and the 3DR Pixhawk (3D Robotics, Berkeley, California, USA) autopilot system was used for autonomous flight between takeoff and landing. Standard stabilization loops and waypoint-following controls were implemented. A ground station computer operated as the command and control station during flight, and it was linked with the air vehicle through a 900MHz wireless transceiver manufactured by 3DR. The Lancaster Mark III Rev 3 (PrecisionHawk, Raleigh, North Carolina, USA), another fixed-wing UAV, was used to determine the utility of a commercially available UAV and data-processing system for such a large research project (Fig 5b). This air vehicle was equipped with an auto-pilot system and two interchangeable multispectral cameras. PrecisionHawk’s proprietary flight control algorithm detects weather conditions at altitude and creates an optimal flight path after a few initial circles in the area of interest. The company provides image pre-processing services such as orthomosaicking and digital surface model (DSM) generation through its cloud-based server. The two fixed-wing UAVs were deployed to cover an entire route pack in one flight, while the rotary-wing UAV, the TurboAce X88 (TurboAce, Orange, CA, USA), was deployed to cover one plot area in a single flight to acquire more detailed information at a lower altitude (Fig 5c). The sensor payload of the X88 was mounted on a gyroscopically stabilized 3-axis gimbal, which minimized the effect of the airframe motion caused by wind.

**Table 3:** Specifications and configurations of the three UAVs used in this study.

	Anaconda fixed-wing	Lancaster fixed-wing	X88 Octocopter
Wingspan (m)	2.0	1.5	0.9
Weight w/o payload (kg)	2.4	1.4	2.5
Maximum payload (kg)	4.0	1.0	2.8
Battery/flight (mAh × number)	5,000 × 1	6,000 × 1	6,600 × 2
Endurance w/ payload (min)	20–30 (45 max)	20–30 (45 max)	8–10
Air speed (m s^-1^)	15	12–15	6
Flying altitude AGL (m)*	120	120	15–20

* Our COA permitted a maximum of 182.9 m flight altitude with a professional pilot on site.

### Sensor Payloads

**Fig 6:**
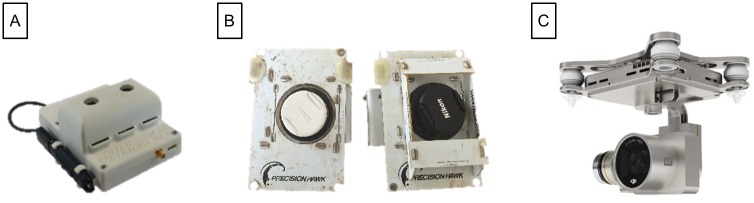
Sensors carried by the UAVs used in this study. (A) Sentek GEMS multispectral camera carried by the Anaconda fixed-wing UAV. (B) Nikon J3 digital camera (left) and modified multispectral camera (right) carried by the Lancaster fixed-wing UAV. (C) DJI P3-005 4K camera carried by the X88 octocopter.

Several sensors (Fig 6) were carried aboard the UAVs. Principally, multispectral cameras and true-color digital cameras were used to collect crop spectral and morphological information (Table 4). A Sentek GEMS multispectral camera system (Saint Louis Park, MN, USA) with integrated GPS and inertial measurement unit (IMU) sensors and onboard image storage capability was used with the Anaconda UAV. The cameras carried (one at a time) by the Lancaster UAV were a true-color Nikon J3 digital camera (Tokyo, Japan) and a color-infrared camera modified from the Nikon J3 digital camera. They were triggered by an onboard controller to vary the frame rate based on flight speed for optimal overlap between images. The X88 octocopter UAV carried a DJI P3-005 4K camera (Shenzhen, China) that was triggered remotely by the operator. Images collected with all three UAVs were geo-tagged with onboard GPS data.

**Table 4:** Sensor configuration used in this project.

	Sentek GEMS multispectral camera	Nikon J3 digital camera	Multispectral camera modified from Nikon J3	DJIP3-005 4K Camera
UAV platform	Anaconda fixed-wing	Lancaster fixed-wing	Lancaster fixed-wing	X88 octocopter
Spectral sensitivity (nm)	Blue: 399–501	True-color	Green: 500–560	True-color
	Green: 502–604	Blue: 420–500	Red: 570–640	Blue: 420–500
	Red: 558–672	Green: 500–560	NIR: 680–760	Green: 500–560
	NIR: 742–878	Red: 570–640		Red: 570–640
Dynamic range	10-bit	12-bit	12-bit	NA
Shutter type	Global shutter	Rolling shutter	Rolling shutter	Global shutter
Exposure time (ms)	Fixed but varied from flight to flight	1/2.0	1/3.2	Fixed but varied from flight to flight
Aperture (f-stop)	NA*	f/1.6	f/3	Fixed but varied from flight to flight
ISO	NA	160	800	Fixed but varied from flight to flight
Imaging sensor pixel resolution (megapixel)	1.2 (1280 × 960)	14.2 (4608 × 3072)	14.2 (4608 × 3072)	12.0 (4000 × 3000)
Frame rate (s/frame)	1.4	Varied based on flight speed	Varied based on flight speed	Triggered manually ~0.3–0.5
Typical flying altitude (m)	120	120	120	15–20
Ground sampling distance (cm)	6.5	1.86	3.44	0.8
Weight (g)	170	244	244	130

* NA, unknown or not available to be configured

### Flight and Sensor Configuration

Most flights were made within 2.0 h of solar noon. Flight and sensor parameters were configured to ensure collection of high-quality images with adequate overlap between images for mosaicking purposes, and to minimize pixel smearing. Parameters selected included (1) flight altitude, speed, and path; and (2) sensor exposure time, aperture, sensitivity (ISO) and frame rate. While low-altitude UAV flights offer much higher image resolution, the ground coverage of an individual image is much smaller, potentially resulting in inadequate coverage and insufficient overlap between images if a low flight speed cannot be maintained. This issue was especially critical with the fixed-wing UAVs, with which most images were collected. The Pix4Dmapper software package (Pix4D SA, Lausanne, Switzerland) was used to mosaic individual images together into one large route-pack image for a given data collection event. Pix4D requires at least 75% forward overlap and 60% side overlap to generate high-quality mosaics. Higher overlaps are preferred for agricultural fields to identify details in the relatively homogeneous visual content in the images. Field breeding trials commonly have different cultivars in adjacent rows (76 cm to 101 cm row spacing), so a low level of mosaicking error is allowable when using UAV images for phenotyping in breeding trials. Increased overlap can be achieved by increasing UAV altitude, reducing UAV speed, increasing the camera’s field of view (FOV), or increasing the camera’s image-acquisition frame rate.

**Fig 7:**
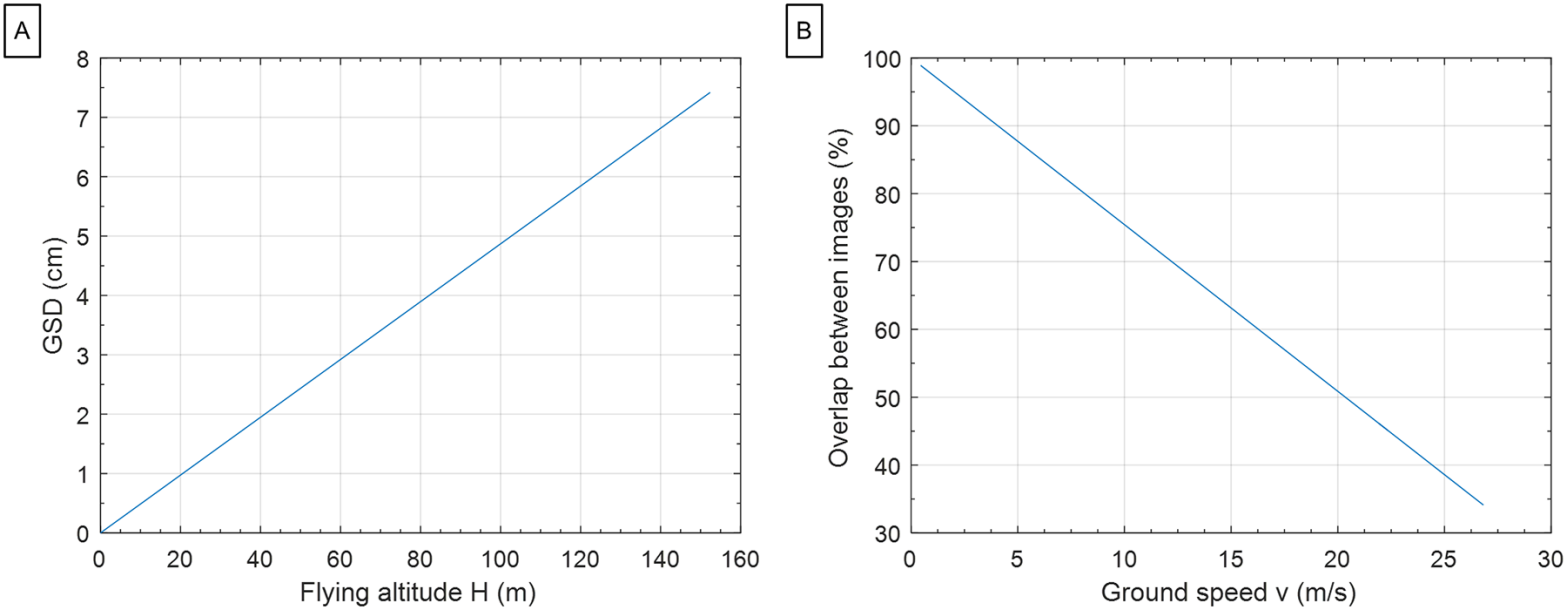
Relationship between flight and sensor parameters used to determine optimal flight and sensor configurations before flights (use Anaconda fixed-wing and Sentek multispectral camera as an example here). (A) GSD and flying altitude AGL under a fixed sensor FOV (26.31° vertically). (B) Image overlap and UAV ground speed under a fixed flying altitude (120 m) and a fixed sensor frame rate (1.4 s/frame).

**Fig 8:**
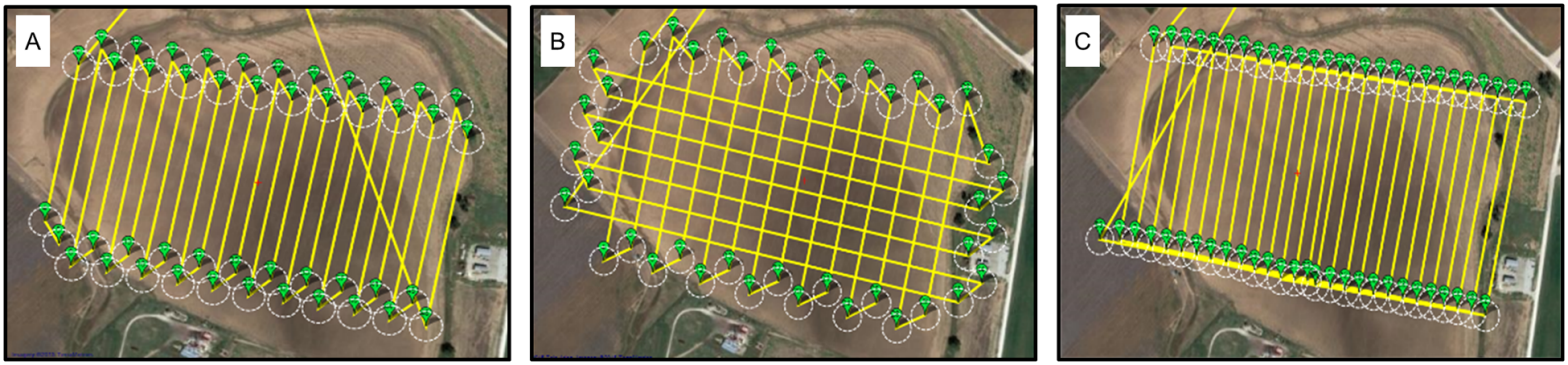
Three different flight paths over a 30-ha route pack evaluated by Anaconda fixed-wing UAV in this study. (A) Standard parallel flight path. (B) Cross-stitch flight path. (C) Moving-box flight path. The yellow lines represent planned flight paths; the green balloon-shape icons represent waypoints along the flight path for GPS navigation.

The FOV and frame rate of the Sentek GEMS multispectral sensor were calculated with Eqs 1 and 2. Ground sampling distance (GSD; i.e., spatial resolution) is a function of flying height above ground level (AGL), *H* (Fig 7a). Overlap between images is a function of UAV ground speed, *v*, assuming fixed values of *H* and sensor frame rate, *fr* (Fig 7b). Based on these relationships, a constant achievable UAV air speed was assumed and entered into the autopilot system to establish the desired image overlap given the selected flying height and expected wind speed and direction. Sufficient side overlap was achieved by a denser flight path. Three different patterns of flight path were tested for the Anaconda fixed-wing vehicle (Fig 8). The ‘moving box’ pattern most easily achieved adequate side overlap in this study due to its smoother (i.e., larger-radius) turns.

$$\{\begin{array}{c} G_{h}=S_{h}\cdot \frac{H}{F}\\ G_{v}=S_{v}\cdot \frac{H}{F} \end{array}$$ (1)

in which *G*_*h*_ and *G*_*v*_ are the horizontal and vertical ground coverage in m, respectively; *S*_*h*_ and *S*_*v*_ are the horizontal and vertical dimensions of sensor’s imaging detector in mm, respectively; *H* is the flying altitude AGL of the UAV in m; and *F* is the sensor’s focal length in mm.

$$fr=\frac{v_{max}}{G_{v}\cdot \left(1-P\right)}$$ (2)

in which *fr* is the sensor’s frame rate in frames/s; *v*_*max*_ is the maximum ground speed of UAV in m/s; *G*_*v*_ is the vertical ground coverage in m; *P* is the percentage of required forward overlap.

Camera exposure time, aperture and ISO settings were fine-tuned and balanced to avoid over- and under-exposure of the crops and to minimize image motion blur (Table 4). Exposure times were fixed for the sensors prior to each flight so that brightness variation between images would depend only on illumination changes during the flight, the imaging angle variation caused by platform tilt, and the albedo variation of the objects in an image. Vigorous vegetation has a much higher reflectance in the NIR band than in RGB bands, so different fixed exposure times were used for the RGB and NIR cameras on the Sentek multispectral camera system to effectively use the dynamic range of the separate imaging detectors.

### Geographic Registration and Radiometric Correction

**Fig 9:**
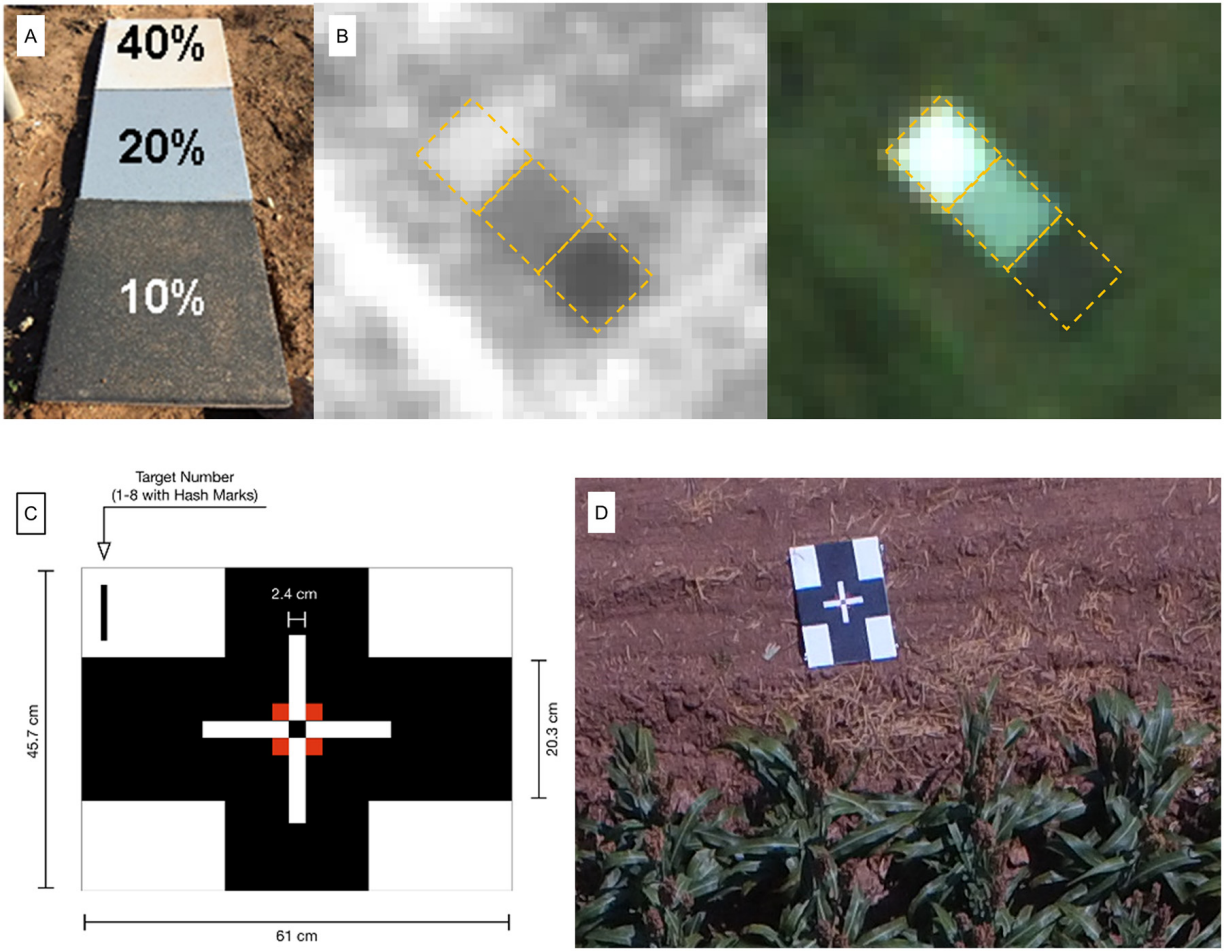
Two types of GCPs were used in this study. (A) A set of semi-permanent painted concrete tiles with 10%, 20% and 40% reflectance for radiometric correction. (B) A set of semi-permanent GCPs as seen in the NIR and RGB images collected with a fixed-wing UAV at 120 m AGL. (C) A portable wooden frame GCP covered with canvas painted with a double-cross pattern. (D) A portable GCP as imaged with the octocopter at 15 m AGL.

Ground control points (GCPs) are critical for geographic registration of images during mosaicking and when overlaying images collected at different times. Some applications also require radiometric calibration references so that reflectance values of image objects can be calculated. In this study, 61 × 61 cm concrete tiles were installed semi-permanently at the corners and interior locations of all route packs where they would not interfere with farm operations. These tiles were used as both GCPs and radiometric calibration references for images collected with the fixed-wing UAVs. Tiles were painted black (≈10% reflectance), dark gray (≈20% reflectance) and light gray (≈40% reflectance) (Fig 9a), and their spectral reflectance was measured *in situ* on a regular basis with a handheld spectrophotometer (FieldSpec, ASD, Boulder, CO, USA). The size of the tiles was selected such that they were clearly distinguishable from the background with at least 6 × 6 pixels in aerial images (Fig 9b). Each semi-permanent tile was measured for position at the beginning of the season with a real-time kinematic GPS receiver to give a precise latitude and longitude of the center of the tile.

Portable GCPs were also distributed around target plots before certain flights. Particularly in the rotary-wing flights, a 47.5 x 61.0 cm wooden frame covered with canvas fabric and painted with a double-cross pattern was used for each flight of the octocopter (Fig 9c and 9d). The locations of these temporary GCPs were measured prior to each flight.

### Data Pre-processing

After a flight, pre-processing was implemented on the raw image data for 1) image correction and format conversion with proprietary software for a specific sensor; 2) ortho-mosaicking with Pix4Dmapper; 3) secondary band registration, if necessary; 4) radiometric calibration to convert pixel values from digital numbers to reflectance; and 5) brightness adjustment to alleviate cloud shadow effect if necessary. Radiometric calibration was implemented with linear relationships established between the aerial measured reflectance and the ground-truth reflectance of the GCPs (Eq 3). Because an entire image mosaic may be compiled from images taken over a period of over 20 minutes, it is possible for clouds to transit across the image area and create cloud shadows. For those flights conducted when many clouds were in the sky, a band normalization algorithm [36] was implemented to adjust the brightness caused by the resulting cloud shadows.

$$r_{\left(x,y,b\right)}=\beta_{1\left(b\right)}\cdot DN_{\left(x,y,b\right)}+\beta_{0\left(b\right)}$$ (3)

in which *r*_(*x*,*y*,*b*)_ is the calibrated reflectance of pixel (*x*,*y*) in spectral band *b*; *DN*_(*x*,*y*,*b*)_ is the digital number of that pixel (*x*,*y*) in spectral band *b* of a mosaic; and *β*_1(*b*)_ and *β*_0(*b*)_ are the slope and intercept of the linear regression model of spectral band *b* developed with the digital numbers of GCPs and their corresponding known reflectance in that spectral band.

True-color images with a 4000 × 3000 pixel array, collected with the rotary-wing UAV, were acquired in JPEG format with minimal compression in favor of an uncompressed image format due to the extensive processing time of structure from motion (SfM) and the large number of images (typically 800 to 1,600, depending on the size of area of interest) required to provide the overlap necessary to generate very high resolution SfM point clouds. The geotags contained in each image’s metadata recorded the approximate position of the UAV at the time of capture; this information was utilized by Pix4D (Pix4D SA; Lausanne, Switzerland) to assist in the process of identifying tie points among overlapping images. The imagery was manually reviewed to identify and discard images that were incidentally taken during takeoff or landing, thus resulting in more consistent spatial resolution. Any images containing motion-induced blur were discarded and images containing hotspots (i.e., areas with a loss of spectral information due to overexposure or sun glint) or significantly different illumination conditions as a result of cloud cover were discarded, when doing so did not reduce image overlap below five images per point.

## Case Studies

The field researchers involved in this project sought to answer multiple biological questions, enabling development of case studies relevant to their specific crops. Several case studies are included here to give a sense of the breadth of the project.

### Case Study 1: Plant height in maize and sorghum

#### Background and objective

Commercial maize cultivars in drought-prone Texas conditions have shown high correlation between height and yield (r^2^ = 0.61; [8]), a fact often not found to be true in less stressful environments. A similar pattern between height and yield has also been observed in sorghum [37]. It is hypothesized that the relationship is based on the plants’ ability to overcome stress throughout the growing season, possibly due to hybrid vigor. Monitoring plant height of hybrids in relation to environmental conditions throughout the growing season will help identify superior parents and genomic regions contributing to this process that can be combined through breeding. Estimating plant height in maize has been attempted with UAV images before [21, 38], and it was found to be challenging due to the "spiky" nature of the plants leaves. While the literature does not include estimating sorghum height from UAV images, similar difficulties are expected due to the visual similarities between sorghum and maize. The objective of this case study was to use UAV remote sensing data to measure plant height in maize and sorghum breeding plots.

#### Experiment design

**Fig 10:**
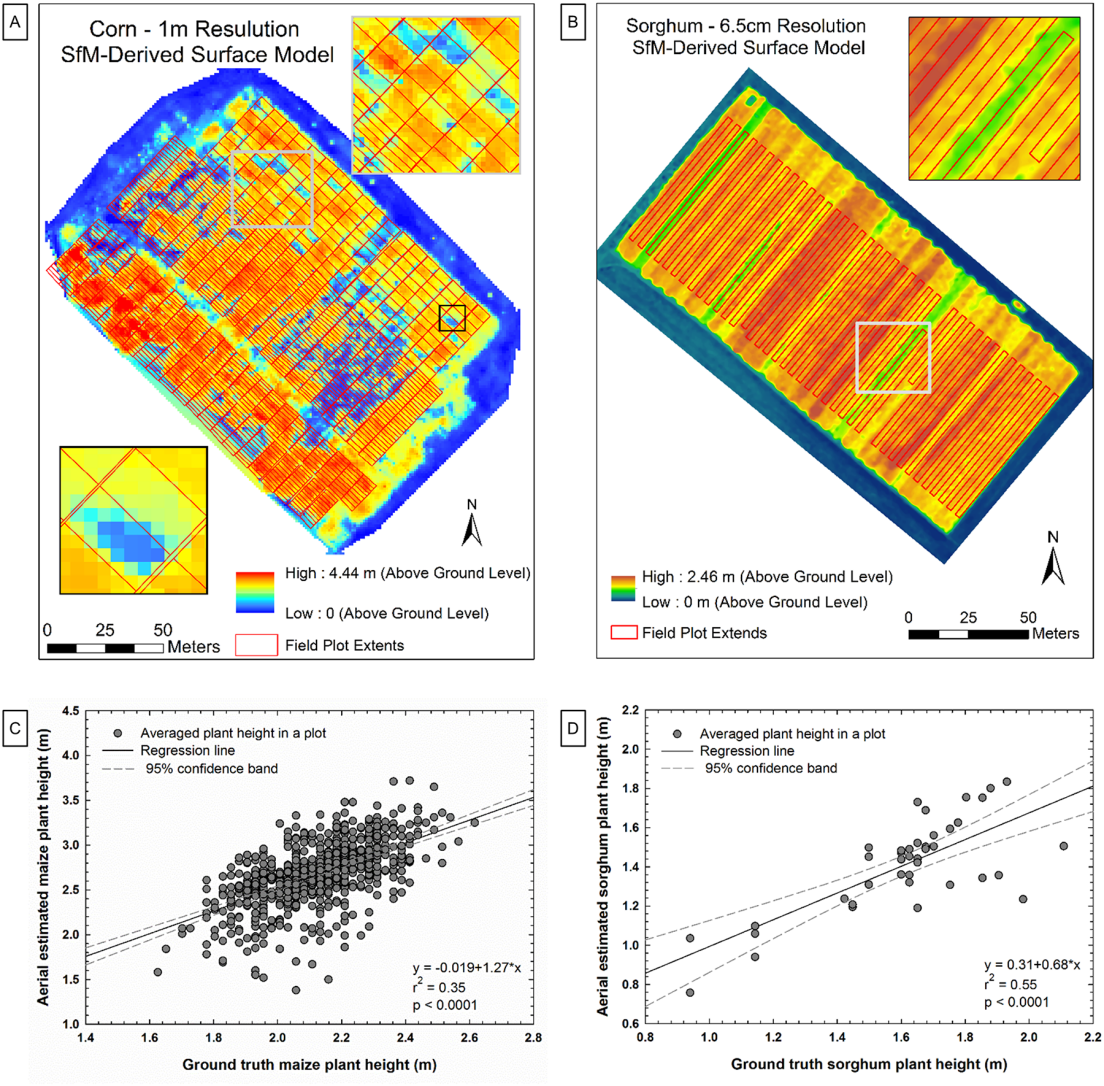
Results of plant height estimates. Digital surface models (DSMs) and correlations between aerial estimated plant height and ground truth plant height on maize (A) based on 705 observations (C), and on sorghum (B) based on 40 observations (D).

Images from a large field of maize were collected with the high-resolution digital camera onboard the X88 rotary-wing UAV on July 22, 2015, a few weeks before harvest. Five separate field trials were completely imaged and three trials were partially imaged. A total of 1065 plots with 351 different breeding hybrids were included along with 12 widely replicated commercial checks, totaling 148 of these plots. A plot in this case was one variety planted in two paired rows 7.6 m long, although one test of 180 plots had five rows in a plot. A digital surface model (DSM) (Fig 10a) was calculated from the image data and used to estimate height. The height estimates were compared to ground truth measurements taken approximately three weeks earlier. Ground truth measurements involved measuring plant height from the ground to the tip of the tassel [8] as well as the number of plants per plot (population count). A genetic ANOVA analysis, to examine the consistency of UAV estimated height across replications in the field was also conducted.

Images from a field of energy sorghum were collected with the multispectral camera onboard the Anaconda fixed-wing UAV on September 5, 2015. Forty genotypes at a late vegetative stage were imaged. The DSM (Fig 10b) was used to estimate plant height, and the height estimates were compared to ground truth measurements taken on September 11. Ground truth measurements involved measuring plant height from the ground to upper leaves or panicle, depending on whether the genotype had flowered at that time.

#### Results and discussion

Across the 1065 maize plots considered, the correlation between UAV estimated plant height and ground truth was significant but weak (r^2^ = 0.11; p<0.0001). The correlation improved for the 180 five-row plots (r^2^ = 0.27; p<0.0001) likely due to larger sampling areas within a plot. One interesting result was that UAV estimated plant height correlated better with population count (r^2^ = 0.23; p<0.0001) than did ground truth plant height across the entire dataset. A close look at the data indicated that a significant number of plots either sustained extensive feral hog damage in the time between ground truth and imaging or they contained less than 15,000 points in the DSM. These plots were removed, and the remaining 705 plots were analyzed as a group. These plots had higher correlation (r^2^ = 0.35; p<0.0001) (Fig 10c) than did the entire data set.

The genetic ANOVA indicated low genetic variance (≈8% of total variation detected) when compared with ground truth height genetic variance (≈40% of total variation). However, both detected a large field spatial component caused by flooding that had occurred early in the growing season. A number of reasons may explain the generally weak correlation between UAV estimated and ground truth plant height. First, the fixed-wing UAV estimates did not have adequate resolution to distinguish the small tassels atop the plants, which were measured on the ground. Second, UAV images were collected about three weeks after ground truth data, and the plants had dried down significantly such that the plant canopy was not as erect as earlier in the season. Third, plot boundaries were manually drawn on the mosaicked image, and there was thus variability in pixel selection accuracy. Finally, there was some elevation variation in the bare ground DSM that was not taken into account in UAV estimates of plant height. Taken together, these issues suggest that process improvements are needed and that future results are likely to be improved.

A stronger correlation between UAV estimated and ground truth height was found with the sorghum plots (r^2^ = 0.55; p<0.0001) (Fig 10d). The energy sorghum plants in this study grow very tall and were commonly in excess of 2.0 m during data collection. Field measurement of tall plants is difficult, and the ground truth height for a row was sampled at one location with a meter stick. Another problem was that some of the plant lines were in the vegetative growth stage, thus having no definitive plant apex, making it difficult to distinguish the top of the plant from a fixed-wing UAV. Thus errors could be expected in both ground truth measurements and UAV estimates. Additionally, the overlap between images was sometimes insufficient to produce a high-quality mosaic due to excessive ground speed caused by tailwinds (ground speed ranged from 20 to 55 mi h^-1^ or 8.94 to 24.6 m s^-1^) and the tilt of the UAV. Furthermore, the multispectral camera provided for a GSD of 6.5 cm at 122 m AGL, which is marginal for identifying individual plant components like a top leaf. A higher resolution camera with a faster frame rate, as well as slower and more stable flights, would improve plant height estimation from aerial images.

The lower flying height of the rotary-wing air vehicle provided for higher resolution images, but with its slower speed it also provided reduced area coverage. In contrast, the fixed-wing air vechicle could cover almost the entire farm in a single day, but its faster speed resulted in less image overlap, reducing the quality of three-dimensional surface modeling. In order to produce high-quality plant height growth curves capable of shedding light on differential growth of varieties under stress [39, 40], both systems would need improvement. Advances in battery life for rotary-wing air vehicles to enable greater coverage, and advances in cameras—faster frame rate, shorter exposure time, and higher resolution—for fixed-wing air vehicles are needed.

### Case Study 2: Winter wheat biophysical properties

#### Background and objective

Remote sensing is effective for estimating crop biophysical properties [41–43], and data from multispectral cameras are commonly converted to descriptive indices like normalized difference vegetation index (NDVI) to estimate crop characteristics from remote sensing data. The objective of this case study was to investigate the use of UAV remote sensing data for developing empirical models to estimate leaf area index (LAI) and percent canopy cover of wheat.

#### Experiment design

**Fig 11:**
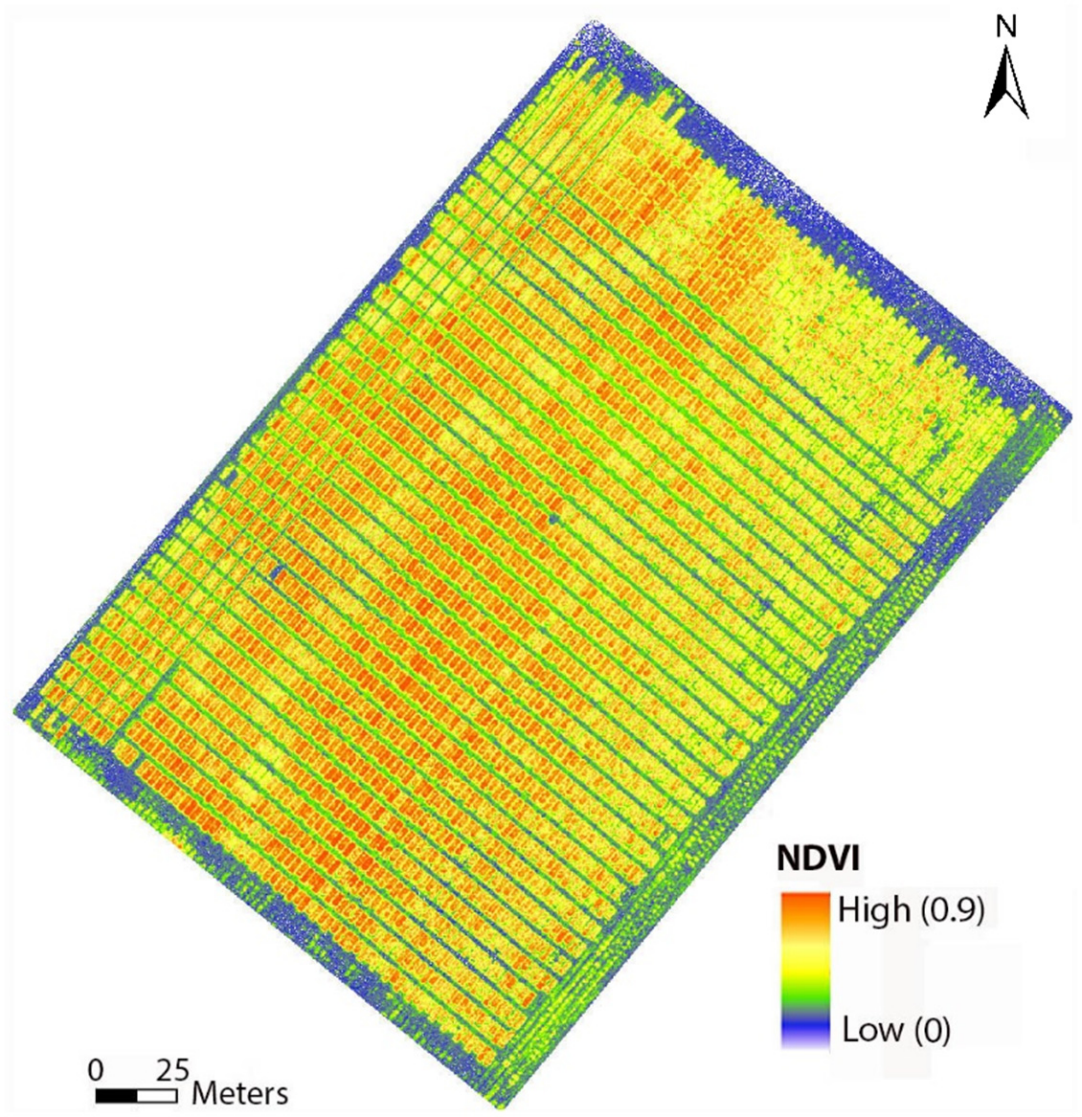
NDVI map generated from multispectral data collected with the Sentek sensor onboard the Anaconda fixed wing UAV platform.

Flights were conducted over a variety trial of winter wheat (Triticum aestivum L) on January 23 and February 26, 2016, with the Sentek sensor on the Anaconda fixed-wing UAV. The field contained a large number of 1.5 x 3.5 m plots, but 40 were used for the biophysical study on the first date and 25 on the second date. Four 2.5 x 10.0-m calibration tarps were laid out in the field for radiometric correction. Reflectance values for the pixels representing each plot were averaged to calculate NDVI (Fig 11), which was compared to ground truth data. Ground-truth canopy cover was estimated from photographs of selected plots collected with a digital camera mounted on a handheld pole looking downward at the crop. Ground-truth LAI was measured with an LAI-2200C plant canopy analyzer (LI-COR, Lincoln, NE) on only the second image acquisition date. NDVI values from bare soil reflectance data were included along with NDVI from plants in creating regression models.

#### Results and discussion

**Fig 12:**
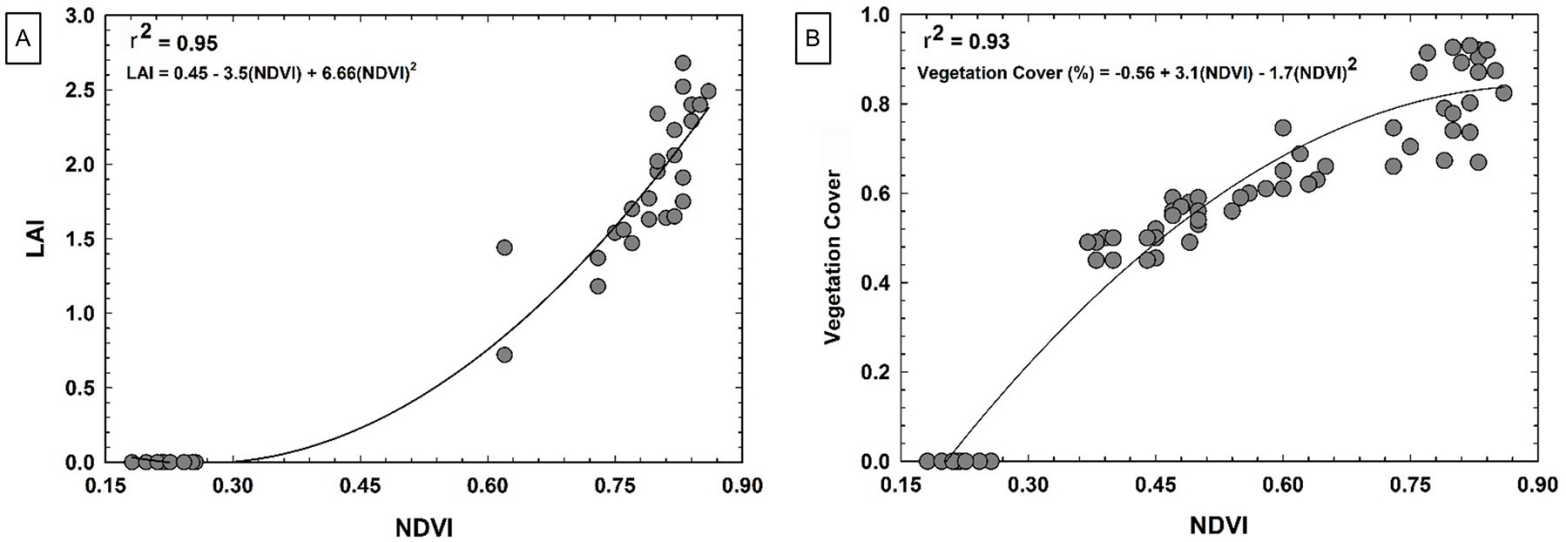
Results of winter wheat biophysical study. (A) Correlation between wheat leaf area index (LAI) measured on the ground using leaf area meter and NDVI calculated from aerial imagery. (B) Correlation between wheat ground cover estimated on the ground and NDVI calculated from aerial imagery.

Strong and statistically significant relationships were found between UAV NDVI and crop biophysical variables (Fig 12). The r^2^ value between UAV NDVI and ground truth LAI was 0.95, based on a curvilinear model. The r^2^ value between UAV NDVI and ground truth percent canopy cover was 0.93, again based on a curvilinear model. These strong relationships suggest that UAV remote sensing data can be used to provide reasonably accurate estimates of LAI and percent canopy cover. A drawback to the empirical models used in this study is that they tend to be site-specific and applicable only to similar situations [44]. However, they can readily provide a ranking of plots on an arbitrary scale, which would often be adequate for the scientific situation at hand; i.e., if used in breeding trials, absolute numbers for LAI and canopy cover may not be necessary, as rankings among plots would be sufficient.

### Case Study 3: Soil and plant interaction

#### Background and objective

Soil variability contributes to high levels of variability in plant environments within most agricultural fields. Soil properties such as clay content are strong drivers of crop growth and set the maximum yield potential through nutrient and water storage capability. Ground-based proximal sensing to estimate soil apparent electrical conductivity (EC_a_) has become common for mapping soil properties in the field (Corwin and Lesch, 2005). Maps of soil variability are valuable to growers in establishing management zones, but these tend to cover broad areas and thus lack detailed data on differences in local environments.

Other factors also affect plant environments in the field, like rainfall distribution and intensity. Data that characterize crop canopy variation during the growing season, like remotely sensed vegetation indices, integrate soil and weather conditions into a spatiotemporal representation of plant response to local environment. The end-of-season representation of plant response to local environment is yield. Thus it is important to consider whether UAV images collected during the growing season can indicate environmental crop stresses that will be borne out in a yield map collected at harvest. If growers were able to link crop stress to environmental factors like soil type, they could potentially develop beneficial site-specific management practices such as variable-rate irrigation. Thus the objectives of this work were 1) to evaluate the relationship between end-of-season crop yield—cotton in this case—and during-season UAV vegetative indices, and 2) to consider how soil properties relate to the expression of environmental crop stress.

#### Experiment design

**Fig 13:**
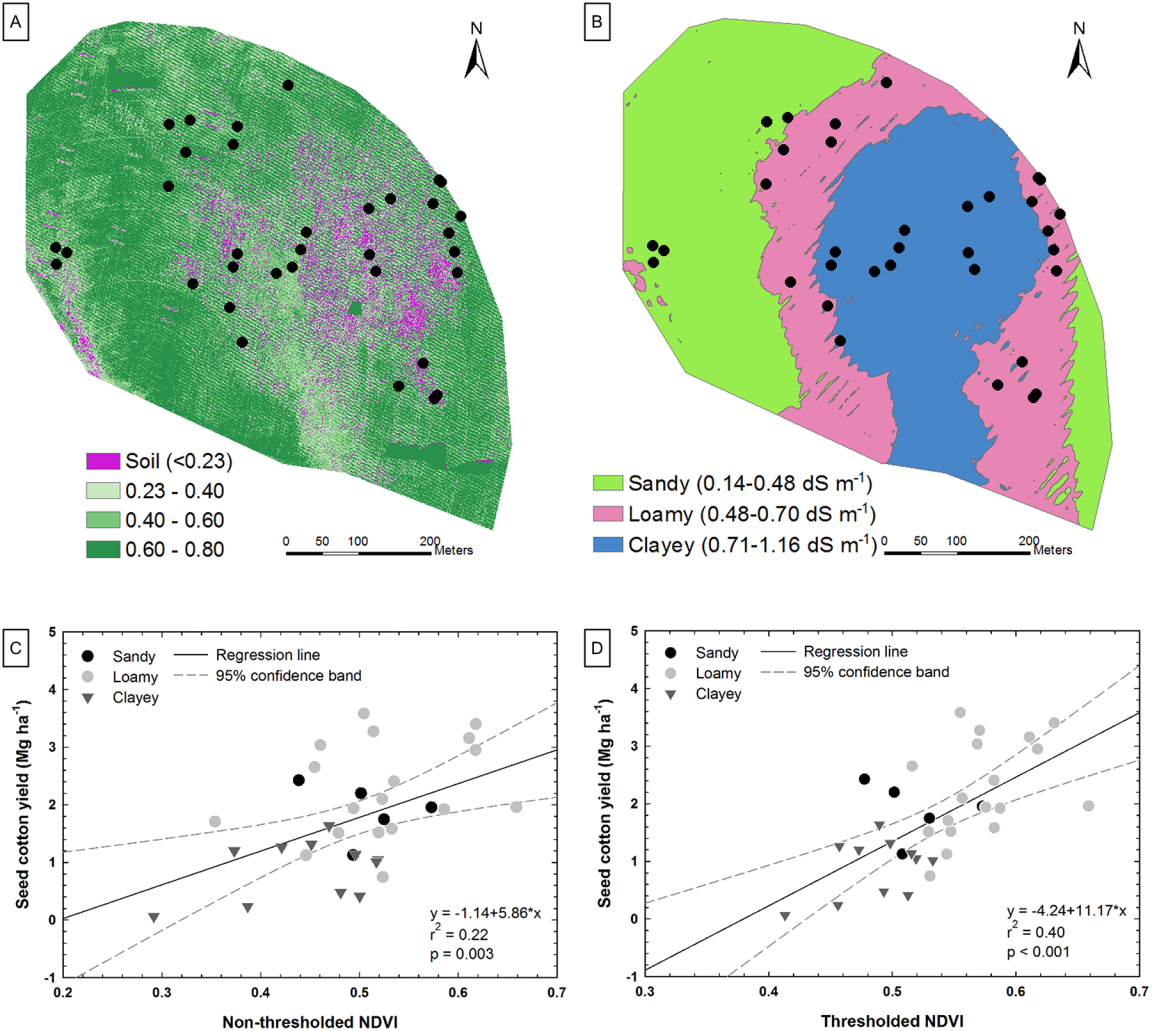
Results of soil and plant interaction study. (A) Normalized difference vegetation index (NDVI) map thresholded to remove bare soil. (B) Apparent electrical conductivity (EC_a_) map of the soil. (C) Correlation between NDVI and seed cotton yield at late growth stage. (D) Correlation between thresholded NDVI and seed cotton yield.

A single cotton variety (Phytogen 499) was planted on a 30 ha field. A survey of soil EC_a_ was conducted with an EM38 (Geonics Limited, Mississauga, Ontario, Canada) instrument when the soil was near field capacity, and the EC_a_ data were classified with K-means clustering to segment the field into predominately sandy, loamy, and clayey textures (Fig 13b). Within each textual group, plant height, stand count and seed-cotton yield were measured for ground truth data.

A set of images was collected on August 26, 2015, a month before harvest, with the Sentek camera onboard the Anaconda fixed-wing UAV. The following indices were calculated from the images: NDVI, Soil Adjusted Vegetation Index (SAVI) [45], and Green Normalized Difference Vegetation Index (GNDVI) [46]. UAV images with 6.5 cm pixels enable differentiation between soil and crop canopy pixels, so thresholding was used with NDVI and GNDVI to exclude bare soil areas from the plant canopy areas (Fig 13a). SAVI and unthresholded GNDVI were compared with thresholded NDVI and GNDVI.

#### Results and discussion

A wet spring resulted in flooding early in the season that led to erosion and ponding after planting and ultimately low stand counts, and later in the season during peak transpiration, little rainfall occurred, reducing yield in the sandier areas. These damaged areas are indicated as bare soil in Fig 13a.

**Fig 14:**
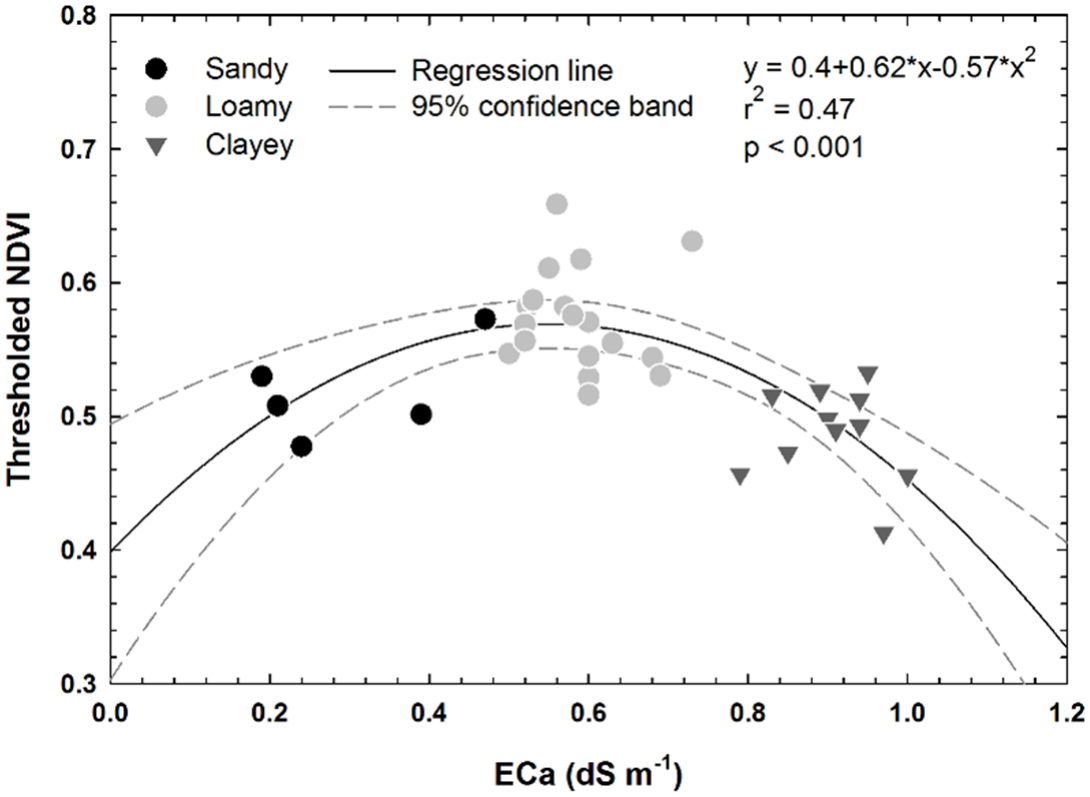
Correlation between thresholded NDVI and soil apparent electrical conductivity (EC_a_).

Moderate correlations were found between SAVI and non-thresholded spectral indices (Fig 13c) and seed-cotton yield (r^2^ = 0.22 to 0.27; p = 0.003). SAVI is supposed to account for soil influence but in this case performed no better than NDVI or GNDVI on the non-thresholded data. Thresholding NDVI (Fig 13d) and GNDVI to remove bare soil pixels from consideration improved r^2^ values to 0.40 and 0.36, respectively (p < 0.001). It was clear that EC_a_ (a proxy for soil environment) had a strong relationship with NDVI (a proxy for crop response) (Fig 14). Medium textured (i.e., loamy) soils have historically had higher yields in this field [47], and again in 2015 they produced the highest yield. While the experiment produced only moderate correlations, it did provide an indication that UAV images can estimate crop stress during the season, and they provide a means to consider the effect of soil variability on local plant environment.

### Case Study 4: Weed management evaluations

#### Background and objective

Weed management is a constant challenge that growers face each year. Particularly important information includes dominant weed species, weed size, crop growth stage, etc. Routine field scouting is important to identify site-specific weed issues and take appropriate control measures, but it is tedious and expensive because large areas need to be covered in a short time, and entering fields cannot be done under wet weather conditions. In rice production, field scouting is even more challenging because long-term flooding can be part of the growth regime. However, UAVs may provide practical solutions to field scouting for weeds. The objective of this case study was to evaluate UAV remote sensing data for assessment of mid-season weed infestations prior to herbicide application.

#### Materials and methods

**Fig 15:**
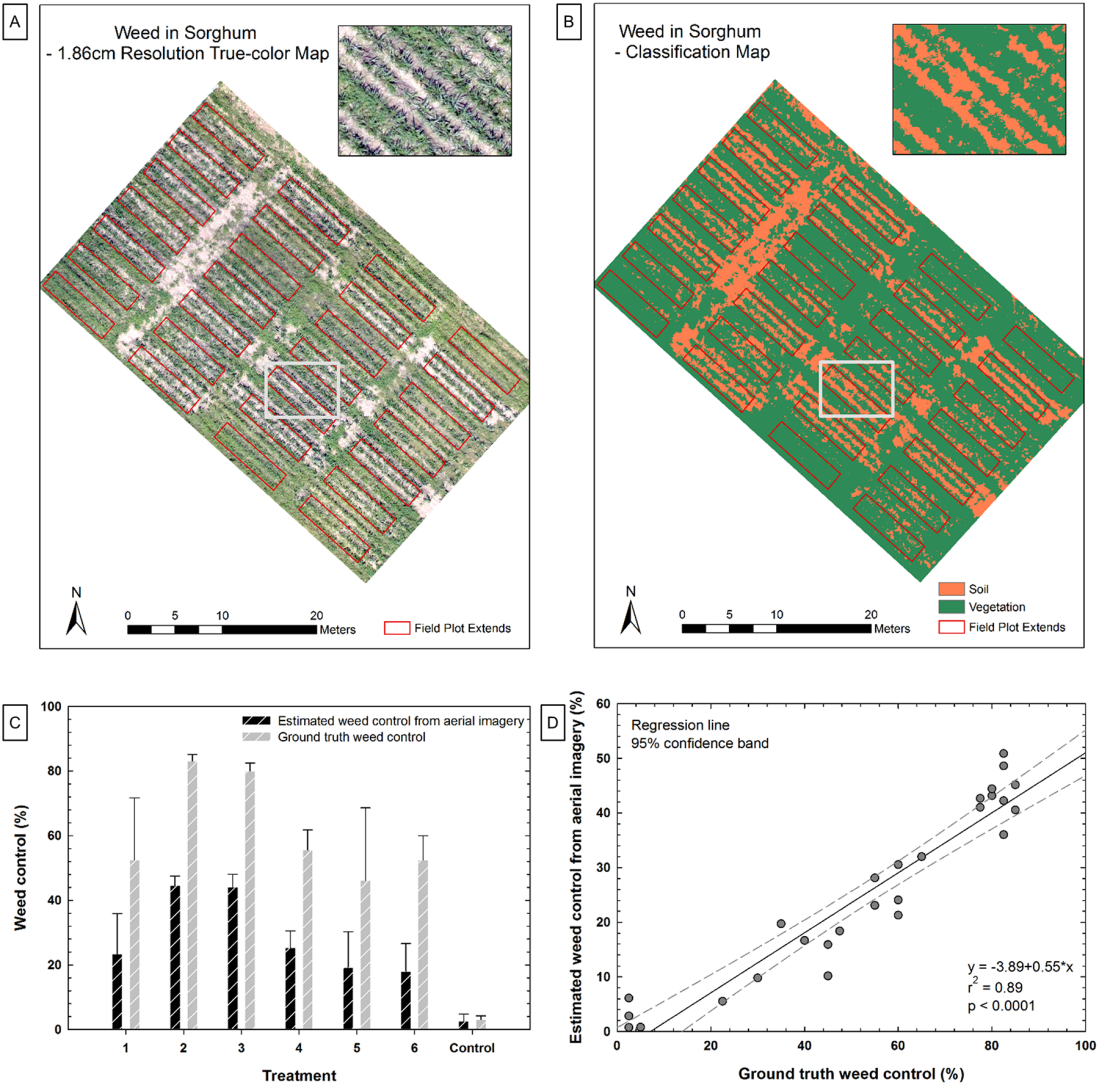
Results of the weed management evaluation study. (A) Aerial image mosaic of a weed management experiment with 28 plots. (B) Classification of soil (brown) and vegetation (green). (C) Comparison between estimated weed control from aerial imagery and ground truth weed control of each treatment. (D) Correlation between estimated weed control from aerial imagery and ground truth weed control based on 28 observations.

Various weed control treatments were applied in a grain sorghum field planted on June 12, 2015. No specific permissions were required to conduct the weed control treatments because the experiment was conducted in the Texas A&M AgriLife Research’s Brazos Bottom Farm. The dominant weed species included Palmer amaranth (*Amaranthus palmeri*), barnyardgrass (*Echinochloa crus-galli*), Texas panicum (*Panicum texanum*), and morningglory (*Ipomoea spp*.). Six herbicide treatments with four replications were used along with a non-treated check. Each plot measured 4 m by 8 m and consisted of four crop rows. Images (Fig 15a) were captured with the true-color camera onboard the Lancaster fixed-wing air vehicle, which flew at 122 m AGL on September 3, 2015. Ground truth was in the form of visual weed control assessments by an expert made on a scale of 0 to 100, with 0 representing no weed suppression compared to the non-treated check and 100 representing complete control (weed-free plot). The Excess Green Index (ExG; [48]) was calculated from the mosaicked image to enhance spectral differences between the vegetation and soil, followed by a K-means classification (Fig 15b). Similar-size regions of interest were created for each plot, including three treated rows and the spaces between them. The ratio of the total count of classified vegetation pixels to the total count of classified soil pixels was calculated for each region of interest as an estimate of weed infestation severity. The sorghum canopy was assumed to be uniform across treatments, and variation in vegetation pixel counts was thus assumed to be caused by weed infestation differences.

#### Results and discussion

A strong and statistically significant correlation was found between UAV estimated weed infestation severity and ground truth weed control (r^2^ = 0.89; p<0.0001; Fig 15d). Significant treatment differences were found in ground truth weed control estimates as well as in the variation of green pixel numbers across the study plots (p<0.0001). One difficulty in differentiating weed from crop was the classification of pixels as exclusively plant or soil material. Improvements in this regard could be approached by increasing image resolution with an improved camera detector, reducing motion blur by slowing the air vehicle or reducing the camera exposure time, or using the spectral features of pixels to determine relative contributions of soil and plant (i.e., sub-pixel analysis). Another difficulty was the lack of weed volume information from the two-dimensional aerial image. Weed scientists usually make application decisions according to several factors including weed coverage and volume. Adding 3D surface model information could be helpful in this regard.

## Conclusions

The first year of this project has enabled a large team of researchers to develop a system of equipment and standard processes for collecting and rapidly analyzing high-quality, low-altitude, aerial images from UAVs over a large research farm. The interdisciplinary nature of the project, while critical to its success, introduced challenges associated with developing shared language and goals. The project demonstrated that integrating UAVs and sensors in such a context is challenging and requires a great deal of planning and coordination, but by the end of the first season, the turnaround time on mosaicked images approached 48 h. Analyses have been conducted for high-throughput phenotyping and agronomic purposes, with some measure of success.

Many breeding and agronomic research programs were involved in this project, and four specific case studies were highlighted. In an effort to develop methods for high-throughput phenotyping with respect to plant height, moderate correlations (r^2^ > 0.50) were found between UAV plant height estimates and ground truth data in research with maize and sorghum. In an effort to relate UAV images to crop biophysical properties, strong correlations (r^2^ > 0.90) were found between UAV NDVI and ground truth LAI and canopy cover. In an effort to relate plant-environment variability to plant-response variability, moderate correlations (r^2^ > 0.40) were also found between UAV NDVI and ground truth seed cotton yield, and soil properties were shown to be indicative of NDVI. In an effort to relate UAV images to weed infestation levels, strong correlations (r^2^ > 0.80) were found between UAV ExG and ground truth visual weed estimates.

## Discussion

### Lessons learned

It would be easy to think that UAV images are merely higher spatial resolution (mm to cm) remotely sensed images, but image data from UAVs must be handled in a different manner than traditional remote sensing data at m-scale resolutions. At such low coverages there is a need to mosaick images together to cover a broader area, and at such high spatial resolutions there is a need to ensure against blurred pixels due to motion. Certain critical sensor criteria are required for providing high-quality image mosaics at high resolution. Camera resolution, along with AGL, limits the theoretical GSD, a fact that is relatively straightforward. Less straightforward is the fact that minimum exposure time limits the actual GSD along the flight path, because at small GSD the air vehicle speed may cover the pixel distance in less time than the camera requires for exposure. This means that pixels may theoretically have a certain small GSD, but they will effectively be “smeared” over a longer distance. Also, camera frame rate along with air vehicle speed limits the amount of forward overlap, which is essential to creating good mosaics. Thus, an ideal UAV camera has high resolution, high frame rate, and short exposure time.

There are also critical air vehicle criteria for providing good images and mosaics. It is necessary that images be captured as close to on-nadir as possible. This situation can be achieved by some combination of a stable air vehicle and a camera gimbal to keep the camera pointed down regardless of air vehicle attitude, but gimbals are not common in small UAS, although one was used on the rotary-wing air vehicle used in this project. Without a gimbal, it is important that the air vehicle remain as level as possible during image capture. Otherwise, geographic registration accuracy in the mosaicking process suffers, and DSM calculations may have unacceptable errors as well. Another safeguard against images that are far off-nadir is to use IMU data to screen out images taken when the air vehicle experienced unacceptable roll, pitch, or yaw. Ideally such a screening process could be automated in software for image pre-processing. Finally, it is common to find at the end of a flying mission that the image overlap was not adequate in certain areas covered. A real-time, during-flight, calculation of the need to re-visit part of the flight coverage area would minimize the possibility of poor mosaics caused by inadequate image overlap.

### Innovations

Certain innovative methods were developed and used in this project. Historically, radiometric calibration has involved laying out on a field a large tarp with varying shades of known reflectance values. The image pixels associated with this target are used to convert pixel values to reflectance values throughout the image. In UAV remote sensing, many images are typically used to make up one large image mosaic. Therefore, it is inadequate to have one small calibration target in a large field that may be imaged over the course of a 30-min. time window. Additionally, the objective of this project involved making numerous flights over the same field, so laying out targets repeatedly, particularly when they are also used as GCPs requiring GPS data to be collected, is not a viable option. Thus semi-permanent tiles were installed throughout each route pack, and the tiles are measured regularly for changes in spectral reflectance.

### Future Needs

The regulatory requirement in the U.S. presents obstacles to regular use of UAVs in agricultural research and production, and a more lenient and permanent regulatory climate is needed for these technologies to flourish fully. Current FAA regulations require that a COA or Section 333 permit be acquired for flying over farms and fields legally. A recent change to FAA regulations requires operators to register the UAVs being flown, in addition to requiring flight training. Project participants need regular updates to keep up with the current regulatory status. This project boasts a large number of faculty, but it also involves numerous graduate students and postdoctoral scholars who will be among the first generation of scientists to use the approaches developed in decision making processes. Developing appropriate training programs for this multi-disciplinary work is critical.

As this project continues into its second year, objectives will transition somewhat from developing flying and data collection protocols to experimenting with new sensors and developing analysis methods that facilitate real world decisions in breeding and agronomics. As goals and aims evolve and grow larger, the importance of developing a shared language among engineers, data processers, and field scientists will continue to be critical to success. Analyzing image data in a way that provides actionable information will be a key goal. One aspect of this involves developing process-based linkages between soil and crop response. Ultimately all the agronomic technologies should be transitioned to decision-support applications for growers and their service providers. With that in mind, the technology and processes can be daunting, so early adopters will have substantial challenges to overcome. The technology must be transferred to the user community in practical ways.

Finally, many image analysis tasks are time consuming, so writing code to automate repetitive processes is a major need in upcoming work. For example, plant breeders working on this project had 38,000 plots in 2015 that had to be delineated by manually drawing polygons on image mosaics, one of the most time consuming activities in the project. These plots were typically adjacent to one another, and plot-level data had to be extracted from them, requiring coordination between data processing experts and field researchers who know where their plots are and the exact regions of interest. Prototype software was developed to automate identification of individual plots as well as radiometric calibration of images, and this software will be further tested in the 2016 growing season. The software is also being designed to assist field scientists in developing image-based metrics like leaf-area index, above ground biomass, and plant height. This software should dramatically increase throughput and decrease error in future studies.

## References

[1] Gerland P, Raftery AE, Ševčíková H, Li N, Gu D, Spoorenberg T, et al World population stabilization unlikely this century. Science. 2014;346(6206):234–7. 10.1126/science.1257469 25301627 PMC4230924

[2] Tilman D, Balzer C, Hill J, Befort BL. Global food demand and the sustainable intensification of agriculture. Proceedings of the National Academy of Sciences. 2011;108(50):20260–4. 10.1073/pnas.1116437108 PMC3250154 22106295

[3] Lal R. Beyond COP 21: Potential and challenges of the “4 per Thousand” initiative. Journal of Soil and Water Conservation. 2016;71(1):20A–5A.

[4] USDA-NASS. USDA National Agricultural Statistics Service: Washington D.C.: USDA-NASS; 2015 [cited 2015 8/16]. Available: http://www.nass.usda.gov/Statistics_by_Subject/index.php?sector=CROPS.

[5] Brummer EC, Barber WT, Collier SM, Cox TS, Johnson R, Murray SC, et al Plant breeding for harmony between agriculture and the environment. Frontiers in Ecology and the Environment. 2011;9(10):561–8.

[6] Duvick DN. Heterosis: feeding people and protecting natural resources. The genetics and exploitation of heterosis in crops. 1999:19–29.

[7] Grassini P, Eskridge KM, Cassman KG. Distinguishing between yield advances and yield plateaus in historical crop production trends. Nat Commun. 2013;4 10.1038/ncomms3918 PMC3905725 24346131

[8] Farfan IDB, Murray SC, Labar S, Pietsch D. A multi-environment trial analysis shows slight grain yield improvement in Texas commercial maize. Field Crops Research. 2013;149:167–76.

[9] White JW, Andrade-Sanchez P, Gore MA, Bronson KF, Coffelt TA, Conley MM, et al Field-based phenomics for plant genetics research. Field Crops Research. 2012;133:101–12.

[10] Araus JL, Cairns JE. Field high-throughput phenotyping: the new crop breeding frontier. Trends in Plant Science. 2014;19(1):52–61. 10.1016/j.tplants.2013.09.008 24139902

[11] Ge Y, Thomasson JA, Sui R. Remote sensing of soil properties in precision agriculture: A review. Frontiers of Earth Science. 2011;5(3):229–38.

[12] Thorp K, Tian L. A review on remote sensing of weeds in agriculture. Precision Agriculture. 2004;5(5):477–508.

[13] Gao B-C. NDWI—a normalized difference water index for remote sensing of vegetation liquid water from space. Remote sensing of environment. 1996;58(3):257–66.

[14] Mirik M, Jones D, Price J, Workneh F, Ansley R, Rush C. Satellite remote sensing of wheat infected by wheat streak mosaic virus. Plant Disease. 2011;95(1):4–12. 10.1094/PDIS-04-10-0256 30743657

[15] Li X, Lee WS, Li M, Ehsani R, Mishra AR, Yang C, et al Spectral difference analysis and airborne imaging classification for citrus greening infected trees. Computers and Electronics in Agriculture. 2012;83:32–46.

[16] Yang C, Odvody GN, Fernandez CJ, Landivar JA, Minzenmayer RR, Nichols RL. Evaluating unsupervised and supervised image classification methods for mapping cotton root rot. Precision Agriculture. 2015;16(2):201–15.

[17] Shanahan JF, Schepers JS, Francis DD, Varvel GE, Wilhelm WW, Tringe JM, et al Use of remote-sensing imagery to estimate corn grain yield. Agronomy Journal. 2001;93(3):583–9.

[18] Serrano L, Filella I, Penuelas J. Remote sensing of biomass and yield of winter wheat under different nitrogen supplies. Crop Science. 2000;40(3):723–31.

[19] Zhang C, Kovacs JM. The application of small unmanned aerial systems for precision agriculture: a review. Precision agriculture. 2012;13(6):693–712.

[20] Sankaran S, Khot LR, Espinoza CZ, Jarolmasjed S, Sathuvalli VR, Vandemark GJ, et al Low-altitude, high-resolution aerial imaging systems for row and field crop phenotyping: A review. European Journal of Agronomy. 2015;70:112–23.

[21] Anthony D, Elbaum S, Lorenz A, Detweiler C, editors. On crop height estimation with UAVs. Intelligent Robots and Systems (IROS 2014), 2014 IEEE/RSJ International Conference on; 2014: IEEE.

[22] Westoby MJ, Brasington J, Glasser NF, Hambrey MJ, Reynolds JM. ‘Structure-from-Motion’ photogrammetry: A low-cost, effective tool for geoscience applications. Geomorphology. 2012;179:300–14. 10.1016/j.geomorph.2012.08.021.

[23] Chapman SC, Merz T, Chan A, Jackway P, Hrabar S, Dreccer MF, et al Pheno-copter: a low-altitude, autonomous remote-sensing robotic helicopter for high-throughput field-based phenotyping. Agronomy. 2014;4(2):279–301.

[24] Hunt ER Jr, Cavigelli M, Daughtry CS, Mcmurtrey JE III, Walthall CL. Evaluation of digital photography from model aircraft for remote sensing of crop biomass and nitrogen status. Precision Agriculture. 2005;6(4):359–78.

[25] Geipel J, Link J, Claupein W. Combined spectral and spatial modeling of corn yield based on aerial images and crop surface models acquired with an unmanned aircraft system. Remote Sensing. 2014;6(11):10335–55.

[26] Bendig J, Bolten A, Bennertz S, Broscheit J, Eichfuss S, Bareth G. Estimating biomass of barley using crop surface models (CSMs) derived from UAV-based RGB imaging. Remote Sensing. 2014;6(11):10395–412.

[27] Berni JA, Zarco-Tejada PJ, Suárez L, Fereres E. Thermal and narrowband multispectral remote sensing for vegetation monitoring from an unmanned aerial vehicle. Geoscience and Remote Sensing, IEEE Transactions on. 2009;47(3):722–38.

[28] Candiago S, Remondino F, De Giglio M, Dubbini M, Gattelli M. Evaluating Multispectral Images and Vegetation Indices for Precision Farming Applications from UAV Images. Remote Sensing. 2015;7(4):4026–47.

[29] Peña JM, Torres-Sánchez J, de Castro AI, Kelly M, López-Granados F. Weed mapping in early-season maize fields using object-based analysis of unmanned aerial vehicle (UAV) images. PLoS One. 2013;8(10):e77151 10.1371/journal.pone.0077151 24146963 PMC3795646

[30] Torres-Sánchez J, López-Granados F, Peña J. An automatic object-based method for optimal thresholding in UAV images: Application for vegetation detection in herbaceous crops. Computers and Electronics in Agriculture. 2015;114:43–52.

[31] Huang Y, Thomson SJ, Lan Y, Maas SJ. Multispectral imaging systems for airborne remote sensing to support agricultural production management. International Journal of Agricultural and Biological Engineering. 2010;3(1):50–62.

[32] Honkavaara E, Saari H, Kaivosoja J, Pölönen I, Hakala T, Litkey P, et al Processing and assessment of spectrometric, stereoscopic imagery collected using a lightweight UAV spectral camera for precision agriculture. Remote Sensing. 2013;5(10):5006–39.

[33] Herwitz SR, Johnson LF, Dunagan SE, Higgins RG, Sullivan DV, Zheng J, et al Imaging from an unmanned aerial vehicle: agricultural surveillance and decision support. Computers and Electronics in Agriculture. 2004;44(1):49–61. 10.1016/j.compag.2004.02.006.

[34] Alchanatis V, Cohen Y, Cohen S, Moller M, Sprinstin M, Meron M, et al Evaluation of different approaches for estimating and mapping crop water status in cotton with thermal imaging. Precision Agriculture. 2010;11(1):27–41.

[35] Yang C, Westbrook JK, Suh CP-C, Martin DE, Hoffmann WC, Lan Y, et al An airborne multispectral imaging system based on two consumer-grade cameras for agricultural remote sensing. Remote Sensing. 2014;6(6):5257–78.

[36] Wu C. Normalized spectral mixture analysis for monitoring urban composition using ETM+ imagery. Remote Sensing of Environment. 2004;93(4):480–92.

[37] Rooney W. Sorghum improvement—integrating traditional and new technology to produce improved genotypes. Advances in agronomy. 2004;83:37–109.

[38] Grenzdörffer G. Crop height determination with UAS point clouds. ISPRS-International Archives of the Photogrammetry, Remote Sensing and Spatial Information Sciences. 2014;1:135–40.

[39] Chen D, Neumann K, Friedel S, Kilian B, Chen M, Altmann T, et al Dissecting the phenotypic components of crop plant growth and drought responses based on high-throughput image analysis. The Plant Cell. 2014;26(12):4636–55. 10.1105/tpc.114.129601 25501589 PMC4311194

[40] Busemeyer L, Ruckelshausen A, Möller K, Melchinger AE, Alheit KV, Maurer HP, et al Precision phenotyping of biomass accumulation in triticale reveals temporal genetic patterns of regulation. Scientific reports. 2013;3. 10.1038/srep02442 PMC3743059 23942574

[41] Rajan N, Puppala N, Maas S, Payton P, Nuti R. Aerial remote sensing of peanut ground cover. Agronomy Journal. 2014;106(4):1358–64.

[42] Ritchie GL, Sullivan D, Vencill W, Bednarz C, Hook J. Sensitivities of normalized difference vegetation index and a green/red ratio index to cotton ground cover fraction. Crop science. 2010;50(3):1000–10.

[43] Zhao C, Li C, Wang Q, Meng Q, Wang J. Automated digital image analyses for estimating percent ground cover of winter wheat based on object features Computer and Computing Technologies in Agriculture II, Volume 1: Springer; 2008 p. 253–64.

[44] Wiegand C, Gerbermann A, Gallo K, Blad B, Dusek D. Multisite analyses of spectral-biophysical data for corn. Remote Sensing of Environment. 1990;33(1):1–16.

[45] Huete AR. A soil-adjusted vegetation index (SAVI). Remote Sensing of Environment. 1988;25(3):295–309. 10.1016/0034-4257(88)90106-X.

[46] Gitelson AA, Kaufman YJ, Merzlyak MN. Use of a green channel in remote sensing of global vegetation from EOS-MODIS. Remote Sensing of Environment. 1996;58(3):289–98.

[47] Stanislav SM. A field-scale assessment of soil-specific seeding rates to optimize yield factors and water use in cotton: Texas A&M University; 2010.

[48] Woebbecke D, Meyer G, Von Bargen K, Mortensen D. Color indices for weed identification under various soil, residue, and lighting conditions. Transactions of the ASAE. 1995;38(1):259–69.

